# The effect of a single closed-circuit rebreather decompression dive in extremely cold water to cardiac function

**DOI:** 10.1007/s00421-023-05392-0

**Published:** 2024-01-08

**Authors:** Laura J. Tuominen, Suvi Tuohinen, Richard V. Lundell, Anne K. Räisänen-Sokolowski, Tomi Wuorimaa

**Affiliations:** 1 Department of Emergency, Emergency Medical Services, Centre for Prehospital Emergency Care, Tampere, Finland; 2 Department of Pathology, Helsinki University, Helsinki, Finland; 3 Centre for Military Medicine, Finnish Defence Forces, Helsinki, Finland; 4 Heart and Lung Center, Helsinki University Hospital, Helsinki University, Helsinki, Finland; 5 DAN Europe Foundation, Finnish Division, Roseto, Italy; 6 Department of Pathology, Helsinki University Hospital, Helsinki University, Helsinki, Finland; 7 Diving Medical Centre, Centre for Military Medicine, Upinniemi, Finland

**Keywords:** Cardiovascular, Echocardiography, Diving medicine, Rebreathers closed circuit, Technical diving

## Abstract

**Purpose:**

Dive-induced cardiac and hemodynamic changes are caused by various mechanisms, and they are aggravated by cold water. Therefore, aging divers with pre-existing cardiovascular conditions may be at risk of acute myocardial infarction, heart failure, or arrhythmias while diving. The aim of this study was to assess the effect of a single decompression CCR dive in arctic cold water on cardiac function in Finnish technical divers.

**Methods:**

Thirty-nine divers performed one identical 45 mfw CCR dive in 2–4 °C water. Hydration and cardiac functions were assessed before and after the dive. Detection of venous gas embolization was performed within 120 min after the dive.

**Results:**

The divers were affected by both cold-water-induced hemodynamic changes and immersion-related fluid loss. Both systolic and diastolic functions were impaired after the dive although the changes in cardiac functions were subtle. Venous inert gas bubbles were detected in all divers except for one. Venous gas embolism did not affect systolic or diastolic function.

**Conclusion:**

A single trimix CCR dive in arctic cold water seemed to debilitate both systolic and diastolic function. Although the changes were subtle, they appeared parallel over several parameters. This indicates a real post-dive deterioration in cardiac function instead of only volume-dependent changes. These changes are without a clinical significance in healthy divers. However, in a population with pre-existing or underlying heart problems, such changes may provoke symptomatic problems during or after the dive.

**Supplementary Information:**

The online version contains supplementary material available at 10.1007/s00421-023-05392-0.

## Introduction 

Diving in arctic water temperatures exposes divers to extreme cold. The low water temperature along with an increased hydrostatic pressure have a significant influence on the cardiovascular system. Hydrostatic pressure acts as a compressive force, increasing central venous return and intrathoracic blood volume accumulation, leading to increased cardiac preload (Epstein 1976, Lin 1984, Gabrielsen 1985/1993) and diuresis (Epstein 1976; Sramek 2000). Increased ambient pressure also elevates oxygen partial pressure, leading to bradycardia, decrease in cardiac output (CO), and triggers increase in systemic vascular resistance (Molenat 2004).

Responses to cold are mainly due to increased peripheral vasoconstriction (Jansky 1996; Alba 2019) and activity of the sympathetic nervous system (SNS) (Sramek 2000). Cold-induced vasoconstriction yet increases centralization of blood flow and thereby cardiac preload. The activation of SNS increases CO; although cold, together with changes in blood shift due to increased pressure, induces an activation of the parasympathetic nervous system (PNS) after the initial SNS activation in cold water (Lundell 2021).

During immersion, alveolar pressure is similar to ambient pressure when a diver is breathing. Yet, a diver must overcome some resistance to ventilate breathing gas mixtures in and out of the lungs, creating a negative airway pressure (Hong 1969; Weenik 2021). This is emphasized especially when diving with a rebreather compared with open circuit diving due to the heavier work of breathing (WOB) (Castagna 2018).

Breathing at increased pressure leads to a greater tissue uptake of inert gasses, nitrogen, and helium. During ascent, tissues may become supersaturated and release the gas to venous circulation in the form of bubbles. Venous gas emboli (VGE) can cause increased pulmonary resistance and subsequently increases the right-side volume load of the heart. There are also indications of bubbles impairing ventricular relaxation (Marabotti 1999).

As a result of these abovementioned mechanisms, even a single dive will contribute to alterations in pressure gradient in the thoracic area, leading to various cardiac and hemodynamic changes. Due to these changes, aging divers with pre-existing cardiovascular conditions may be at risk of acute myocardial infarction, heart failure, or arrhythmias (Asmul et al. 2017; Lippman 2020; Buzzacott 2021). Divers with hypertension are at an increased risk of pulmonary edema especially when diving in cold water (Castagna 2017). In fact, cardiovascular diseases have been recognized as the main cause of diving-related fatalities in up to 20–31% of cases (Denoble 2008; Casadesus 2019; Buzzacott 2021).

The aim of this study, in contrast to previously published research, was to assess cardiac function in a new setting where Finnish technical divers are exposed to two major cardiovascular stressors, extreme cold and pressure. Combining a deep decompression closed-circuit rebreather (CCR) dive with extremely cold water temperature may have a significant effect on cardiac function. Previous studies have been made with recreational dives and/or in warm waters or in hyperbaric chambers.

## Methods

### Study design

The test dives occurred during three weekends in January 2020 and March 2022 (one weekend was postponed to 2022 due to the COVID pandemic) at the old water-filled mine in Ojamo (Lohja, Finland). Thirty-nine experienced, healthy subjects, male (*n* = 35) and female (*n* = 4), participated in the tests. The subjects were recruited from the Finnish recreational technical diving community (*n* = 36) and Finnish Navy (*n* = 3). Each diver performed one dive. All subjects participated voluntarily and gave their informed consent for the study. Each subject filled out a health survey, and a diving physician performed a fit-to-dive examination on the morning of the dive.

The study adhered with the Declaration of Helsinki. Ethical approval was granted by the Ethical Committee of Helsinki University Hospital (HUS/976/2019). Research permission was received from both Helsinki University Hospital (HUS/151/2022) and the Finnish Defense Forces Logistics Command (AP22409, 18.12.2019).

### Preparations and diving protocol

No alcohol was allowed for 24 h before the dive. During the diving day, subjects were instructed to hydrate according to their regular routines until 2 h before the dive. Thereafter, only 5 dl of sports drinks (Gatorade, PepsiCo, Nordic Finland Ltd, Helsinki, Finland) was consumed. Preparations for the dives were made in a room with constant air temperature (19 °C).

Diving conditions were normal for this time of year in Finland: the water was covered with a thin layer of ice, water temperature was 2 °C near the surface and 4 °C at a depth of 45 m. Divers used their own diving equipment during the tests, including their usual undergarments and dry suits. All subjects used their own CCR unit [JJ-CCR (*n* = 28), Megalodon (*n* = 1), rEVO (*n* = 6), AP Inspiration evolution (*n* = 2), Sentinel (*n* = 1), T-Reb (*n* = 1)].

**Fig. 1:**
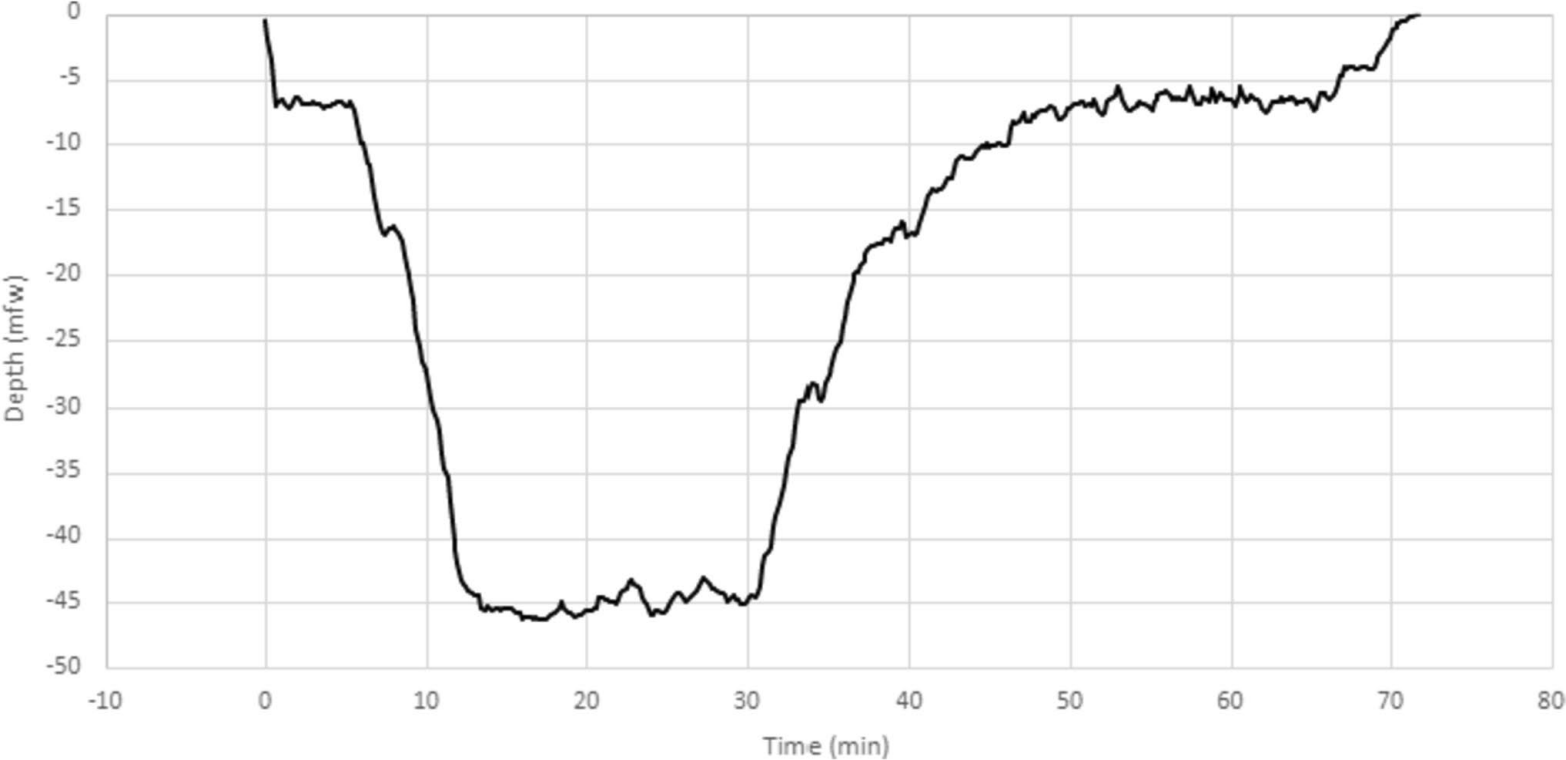
An example of a dive profile with maximum depth of 46.3 mfw and dive time of 73 min

All divers performed identical dives by following a preset line to the bottom depth of 45 m and followed an earlier defined decompression profile: Suunto Fused™ RGBM 2 (Suunto Ltd, Vantaa, Finland) with personal adjustment + 2 (Suunto EON Core and Suunto D5 dive computers). All CCR devices used the same diluent trimix 20/40, and the oxygen controllers maintained constant oxygen partial pressure in breathing loop (pO_2_ 70 kPa in the beginning of dive and pO_2_ 120 kPa at the bottom depth and during ascent). With this set up, the mean total dive time was 64 min. The dive profile of the study is presented in Fig. 1.

### Measurements

Subject demographics were measured with InBody 720 composition analyzer (Biospace Ltd, Seoul, South-Korea). Urine samples were analyzed with a hand-held refractometer (RETK-70 Clinical Refractometer, Tekcoplus Ltd., Hong Kong) to assess the density of the urine after 2-h-controlled hydration with 500 ml isotonic Gatorade drink just before the dive. The second measurement was performed within 30 min after the dive. The divers were allowed to use *p* valves during the dive but were instructed to shut the valves 30 min before surfacing.

### Echocardiographic parameters

Examinations were made by a single cardiac sonographer, clinical physiologist, and diving physician (TW), using two-dimensional (2D) and M-mode echocardiography associated with pulsed- and continuous-wave Doppler (GE Vivid i, GEMS ultrasound, Tirat Carmel, Israel. Cardiac application module H45021JM, transducer 2D 3S-RS with 1.7 – 4.0 MHz broadband multi-frequency range) with a transthoracic approach. A reference examination was performed the same day, 1–2 h prior to the dive. The post-dive examination was performed at 20–40 min after the dive. Echocardiographic measurements of right- and left-sided cardiac chambers and systolic function, along with evaluation of diastology and filling pressure, were performed according to recommendations (Lang et al. 2015; Luis et al. 2019; Mitchell et al. 2019; Rudski et al. 2010). Please see supplementary data for details.

As evaluation of the left ventricular diastology is a complex task involving multiple parameters, grading of the diastology was performed according to recommendations (Ishizu 2008; Nagueh 2016; Oh 2011). Diastolic grade was evaluated either as normal, as Grade 1 (relaxation disturbance), as Grade 2 (pseudonormal filling) or as Grade 3 (restrictive filling) diastolic disturbance using a combination of mitral inflow measurements, isovolumetric relaxation time (IVRT), pulsed tissue Doppler early diastolic velocity (*e*’), and its ratio to mitral inflow *E*-wave (*E*/*e*’). We also calculated the myocardial performance index (MPI), an estimate of combined systolic and diastolic LV function. (Tei et al. 1995).

### Venous gas emboli detection

The presence of VGE in the cardiac chambers was determined with a 2D echocardiographic probe by TW, LT, and RL. Monitoring was performed at 15, 30, 60, 90, and 120 min after surfacing. At all time points, the measurement was made after 1-min rest, during Valsalva maneuver and during arm and leg flexion–extension movement. The observation was recorded and verified with at least one additional observer. Obtained images were graded from 0 to 5 according to the modified method described by Brubakk and Eftedal (Brubakk 2001). The divers were divided into two groups, according to VGE grade, to assess the effect of bubbleload on cardiac function: Grade 0–2 referred to as non-bubbler and Grade 3–4B referred to as bubbler (4B was the maximum bubble grade found after the dives).

### Statistics

We present the variables using medians and interquartile ranges (IQRs). The echocardiographic measurements were divided into pre-dive and post-dive groups. The groups were compared with Wilcoxon-signed rank tests, as some of the data were not normally distributed. The differences between bubblers and non-bubblers were tested using Mann–Whitney *U* tests for the same reason. *p* values below 0.05 were considered significant. All analyses were done using IBM SPSS Statistics version 27 (IBM Corp, Armonk, NY, USA).

## Results

Thirty-nine CCR divers (four female, thirty-five male) completed the decompression dive as planned. After diving and the following day controlled, none of the divers presented any symptoms suggesting a diving incident. Median age of the divers was 43 years (IQR 40–49 years), and they had long diving experience, the median experience was 14.5 years (IQR 11–25 years). Four of the thirty-nine divers had a pre-existing medical condition affecting the cardiovascular system: three had controlled hypertension, but only two had medication for it (olmesartan and losartan), and one had a history of supraventricular tachycardia, which had been successfully treated with ablation. Median body mass index was 26.6 (IQR 24.5–28.22).

During a 64 min dive (IQR 58–70 min), a median weight loss was 0.9 kg (IQR 0.5–1.1 kg) and a median urine density decrease was 0.004 g/mL (IQR 0.001–0.006 g/mL).

**Fig. 2:**
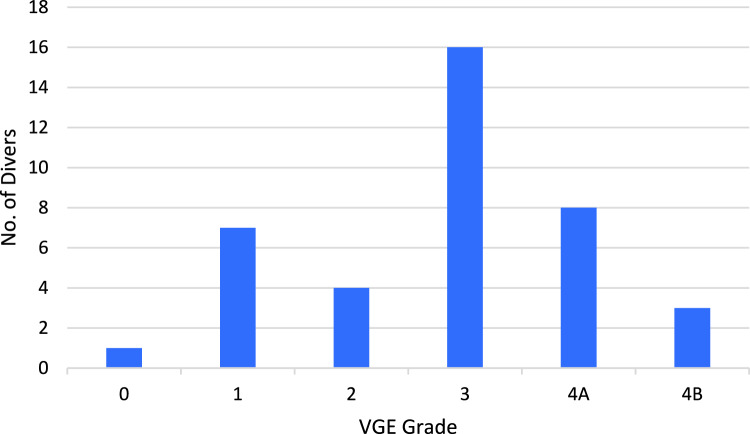
The maximum echocardiographic VGE grading per diver

Venous inert gas bubbles were detected in all divers except for one during the 120-min follow-up. Two divers, with no history of DCI, expressed a few occasional bubbles on the left side, also. The results of the maximum echocardiographic VGE grading per diver are presented in Fig. 2.

### Structural and systolic changes

The changes of the cardiac structures and systolic function pre- and post-dive are displayed in Table 1. Overall, a significant decline was observed in left atrial size by 1.5% [IQR—5.5–0.0%] (*p* = 0.015) and a significant increase in left ventricular end-systolic size by 2.9% [IQR 0.0–6.4%] (*p* = 0.049). A worsening of left ventricular systolic function was seen in all measured parameters. A decline in left ventricular ejection fraction, cardiac output, stroke volume, MAM, S prime and flow rate was observed in 22 (56.4%), 28 (71.8%), 26 (66.7%), 19 (48.7%), 21 (53.8%), and 31(79.5%) of the divers, respectively. Likewise, right ventricular systolic parameters were impaired after the dive as shown in Table 1. A decline in tricuspid annular plane systolic excursion (TAPSE), pulmonary flow, and right ventricle S prime was observed in 22 (56.4%), 28 (71.8%), and 20 (51.3%) divers, respectively. The heart rate did not change after the dive compared to the baseline. Bubblers versus non-bubblers had similar changes in systolic function parameters. The comparisons between bubblers and non-bubblers are presented in Table 2.

**Table 1:** The changes of the structures, systolic and diastolic function pre- and post-dive

Variable	Median change	Baseline	Post dive	*p* value
*Structures*				
LA	− 2.5%	35.0 (33.0−37.5)	34.0 (31.5–36.0)	**0.01**
LVEDD	0.0%	53.0 (50.0–55.5)	52.0 (50.0–55.0)	0.68
LVEDD index	0.3%	26.14 (24.02–27.14)	26.13 (24.21–26.95)	0.53
LVESD	2.9%	33.0 (31.0–35.5)	33.0 (31.0–36.0)	**0.05**
LVESD index	3.2%	16.48 (14.87–17.02)	16.68 (15.08–17.63)	**0.006**
LVLd A4C	− 1.0%	94.0 (89.5–99.0)	93.0 (89.0–100.0)	0.08
LVLd A2C	0.0%	92.0 (88.0–98.0)	91.0 (87.5–99.0)	0.17
LVOT	0.0%	23.0 (22.0–25.0)	23.0 (22.0–24.5)	0.82
Ao asc	0.0%	31 (29–32)	30 (29–32)	**0.03**
IVC	− 5.3%	20.00 (18.50–22.50)	18.00 (17.25–21.00)	0.40
IVS	0.0%	9.0 (8.5–10.0)	9.0 (9.0–10.0)	0.53
*Systolic function*				
HR	0.0%	70.0 (64.5–78.5)	71.0 (59.0 – 80.0)	0.25
EF	− 1.5%	67 (65–69)	65 (62–69)	**0.02**
ET	2.7%	274.0 (255.0–292.5)	280.0 (261.5–294.0)	**0.002**
LV CO	− 7.3%	6.74 (5.88–7.49)	6.14 (5.53–7.05)	**0.004**
LV CO index	− 7.0%	3.26 (3.04–3.76)	3.04 (2.74–3.48)	**0.005**
LV SV	− 3.3%	94.50 (87.50 – 107.50)	92.50 (81.25–106.50)	**0.04**
LV SV index	− 2.9%	46.67 (42.24–53.85)	44.96 (40.03–52.71)	**0.05**
MAM	0.0%	15 (13–16)	14 (12–15)	** < 0.001**
SS'	− 12.5%	7.0 (6.5–8.0)	7.0 (6.0–7.0)	**0.003**
Flow rate	− 8.5%	0.340 (0.320–0.430)	0.335 (0.290–0.400)	**0.003**
RS	− 5.6%	13 (12–15)	13 (12–14)	0.06
RVOT max	− 8.5%	0.79 (0.74–0.88)	0.74 (0.69–0.82)	** < 0.001**
RVOT time	− 4.3%	130.0 (115.5–140.5)	125.0 (110.5–138.0)	**0.02**
TAPSE	− 3.7%	25 (23–27)	24 (22–26)	**0.003**
MPI	8.5%	0.60 (0.48–0.67)	0.63 (0.55–0.74)	** < 0.001**
*Diastolic function*				
IVRT	14.6%	92.0 (71.0–101.5)	99.0 (85.5–115.0)	** < 0.001**
MV E	− 13.2%	0.76 (0.68–0.89)	0.69 (0.58–0.78)	** < 0.001**
MV E Dect	8.2%	188.0 (175.5–195.0)	211.0 (190.5–239.5)	** < 0.001**
MV A	− 8.0%	0.62 (0.54–0.71)	0.59 (0.50–0.66)	0.06
MV E/A	− 8.6%	1.20 (1.06–1.40)	1.10 (0.95–1.48)	**0.02**
SE'	− 11.1%	11.0 (9.0–12.0)	10.0 (8.0–10.5)	** < 0.001**
E/E'	− 2.8%	7.00 (6.13–8.23)	7.30 (6.00–8.31)	0.78
SA'	0.0%	10.0 (8.5–11.0)	10.0 (8.5–11.0)	0.21
LA	− 2.5%	35.0 (33.0–37.5)	34.0 (31.5–36.0)	**0.01**
RE'	− 8.3%	14.0 (12.0–16.0)	13.0 (11.0—14.5)	**0.02**
RA	0.0%	14 (12–15)	13 (12–16)	0.77

**Table 2:** The comparisons between bubblers and non-bubblers

Variable	VGE Grade 0–2	VGE Grade 3–4B	*p* value
EF	− 2.00 (− 7.25; 0.00)	− 1.00 (− 5.00; 1.00)	0.31
ET	8.50 (2.50; 17.25)	6.00 (− 2.00; 26.50)	0.92
LV CO	− 0.42 (− 0.90;−0.02)	− 0.48 (− 1.11; − 0.05)	0.84
LV CO index	− 0.20 (− 0.43; 0.00)	− 0.23 (− 0.54; 0.03)	0.77
LV SV	− 3.00 (− 12.25;−1.50)	− 4.00 (− 9.75; 4.75)	0.40
LV SV index	− 1.27 (− 5.53;−0.58)	− 1.69 (− 4.43; 2.56)	0.33
MAM	0.00 (− 1.25; − 0.00)	− 1.00 (− 2.00; 0.00)	0.64
SS'	− 0.50 (− 1.25; 0.00)	− 1.00 (− 1.00; 0.00)	0.96
Flow rate	− 0.03 (− 0.05;−0.02)	− 0.03 (− 0.05; 0.00)	0.57
MPI	0.60 (0.47; 0.67)	0.63 (0.54; 0.74)	0.13
RS'	− 0.5 (− 2.0; 1.0)	− 1.0 (− 1.0; 0.0)	0.94
RVOT max	− 0.02 (− 0.08; 0.02)	− 0.09 (− 0.12;−0.03)	0.20
RVOT time	− 3.00 (− 19.50; 1.25)	− 6.00 (− 14.50; 2.50)	1.00
TAPSE	− 1.00 (− 3.25; 0.00)	− 1.00 (− 2.00; 0.00)	0.43
IVRT	13.50 (9.75; 18.25)	11.00 (6.00; 20.00)	0.75
MV E	− 0.14 (− 0.18;−0.07)	− 0.10 (− 0.14;−0.04)	0.43
MVE Dect	14.50 (8.50; 37.25)	18.00 (4.50; 50.50)	0.90
MV A	− 0.06 (− 0.08; 0.07)	− 0.06 (− 0.11; 0.04)	0.60
MV E/A	− 0.04 (− 0.23; 0.00)	− 0.11 (− 0.19; 0.06)	0.80
SE'	− 1.0 (− 2.0; 0.0)	− 1.0 (− 2.0;−0.5)	0.42
E/E'	− 0.45 (− 1.05; 0.12)	− 0.10 (− 0.50; 1.00)	0.14
SA'	0.00 (− 1.25; 1.00)	− 1.00 (− 1.00; 0.50)	0.59
LA	0 (− 1; 1)	− 1 (− 2; 0)	0.13
RE'	− 1.0 (− 2.0; 1.0)	− 1.0 (− 4.0; 0.5)	0.63
RA'	− 0.50 (− 2.00; 0.25)	0.00 (− 1.00; 2.00)	0.10

### Diastolic function

**Fig. 3:**
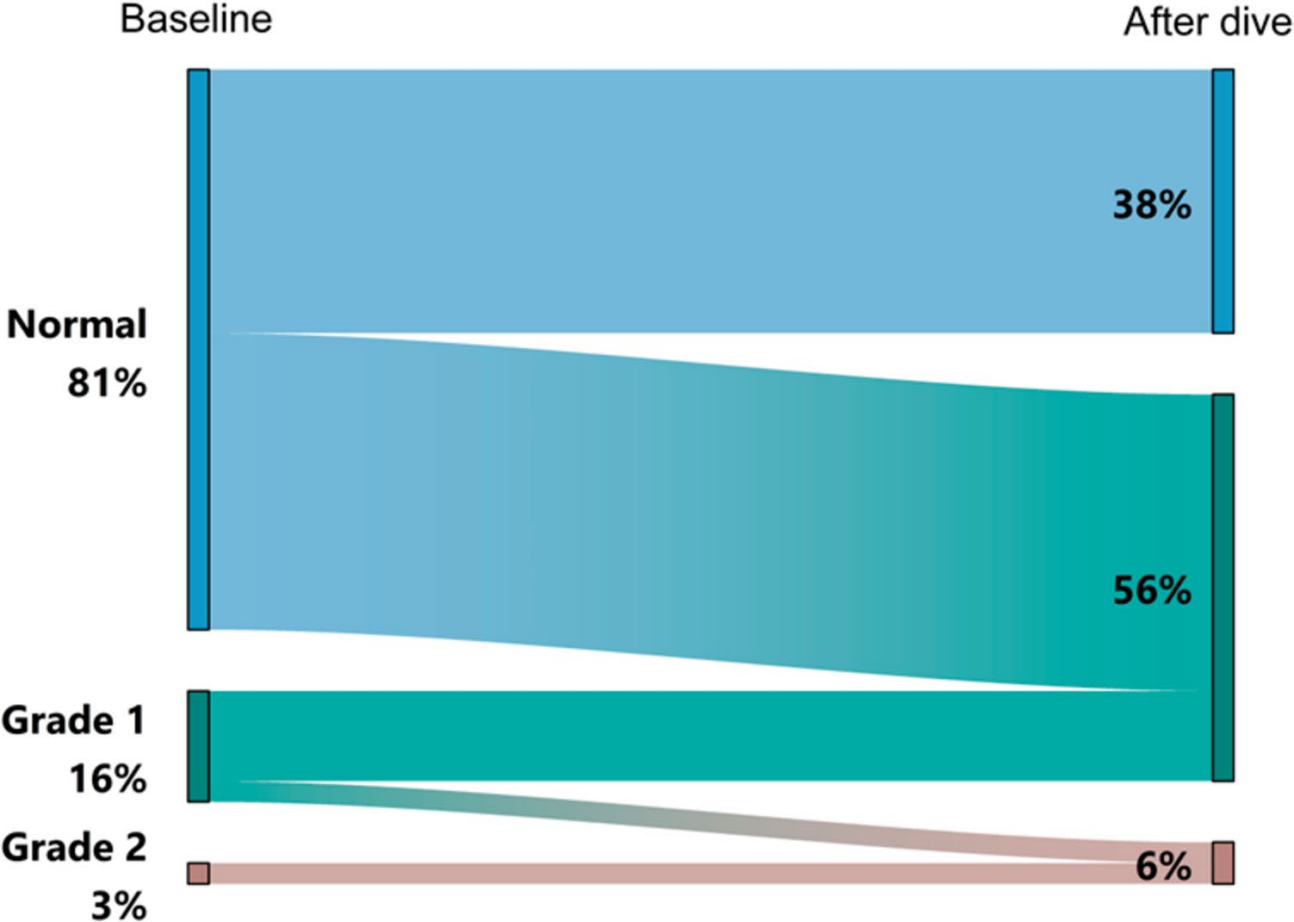
Diastolic function at baseline and after diving

Several diastolic parameters changed significantly from baseline to after diving as shown in Table 1. In the whole group, a significant decrease in mitral E-wave and E/A ratio (*p* < 0.001 and *p* = 0.022, respectively) and a significant increase in mitral declaration time and IVRT (*p* < 0.001 both) imply a diastolic change toward diastolic dysfunction with relaxation disturbance. Diastolic function at the baseline and after diving is displayed in Fig. 3. At the baseline, 32 (82.1%) of the divers had normal diastolic function, 6 (15.4%) had Grade 1, and 1 (2.6%) had Grade 2 diastolic disturbance of the left ventricle. After a dive, diastolic grades deteriorated in 18 (46.2%) divers (*p* < 0.001). The change in weight was similar in divers with and without change in diastolic function (*p* = 0.929).

Furthermore, changes in systolic function were not significantly different in divers with impaired diastolic function except for LV stroke volume indexed value (*p* = 0.041). The changes in the diastolic parameters were not different in bubblers versus non-bubblers.

MPI increased significantly (*p* < 0.001), indicating deteriorating cardiac performance. The increase was seen in 29 divers (78%).

## Discussion

The major finding in our study is that both systolic and diastolic function degraded after a single trimix CCR decompression dive in arctic cold water. It is a novel and noteworthy finding that all the parameters declined, although the changes were subtle. Compared to previous studies involving trimix or CCR divers, our data were concordantly pointing toward impaired cardiac performance. There are previous studies reporting somewhat similar results, also after recreational dives (Marabotti et al. 1999, Marabotti 2013a, 2013b, Boussuges et al. 2006). Other previous studies show varying results and therefore we designed our research in a way that two major diving-related stressors to the cardiovascular system, extreme cold and pressure, would play a great role in our research. We emphasize that our study is conducted in actual diving conditions, where divers regularly perform deep and long dives, though dives in this study were rather short. Despite the selection of divers and rough conditions in this study, the results apply also in warmer conditions, though in lesser effect. Therefore, the results in this research could be generalized including also recreational divers with wet suits (in more tolerable waters).

### Systolic changes after diving

Diving is known to impact cardiac systolic function. Several authors have reported that scuba diving has a depressible effect to the left ventricle (Dujic et al. 2006; Boussuges et al. 2006, 2009; Marinovic et al. 2010) that can return to baseline within 24 h, even after a deep dive (Marinovic et al. 2009), although the same group reported the parameters not returning to baseline 48–72 h after a single air dive (Obad et al. 2007). In turn, Marabotti et al. (2013a; b) and Castagna et al. (2017) reported a preserved left heart function. With small sample sizes and such minor changes in cardiac measurements, the results can vary, as seen in previous studies.

The divers of this study showed a decline of the systolic function after the dive. Even though the changes were clinically insignificant, the fact that all systolic parameters declined is novel and noteworthy. As the divers experienced 0.9 kg loss of weight corresponding to a volume depletion by 0.9 L on average, this may have affected volume-dependent parameters, such as left ventricular ejection fraction, cardiac output, stroke volume, and TAPSE. However, the left ventricular end-systolic, and not end-diastolic, dimension changed, which is in contradiction with volume-dependent changes only. Furthermore, volume-independent systolic parameters, such as flow rate and myocardial pulsed Doppler systolic velocity, declined as well. Therefore, it is evident that the divers experienced a real worsening of the cardiac systolic function after the dive.

One suggested factor interfering with systolic function, cardiac relaxation, and especially heart rate, could be hyperoxia, which has been seen in normobaric and hyperbaric research (Mak et al. 2001; Molenat et al. 2004; Boussuges et al. 2007). It is likely that decompression diving and changes in preload and afterload would cause stress to the diver’s homeostasis, negatively affecting heart function. Yet the exact cause for the systolic changes remains unclear.

### Diastolic changes after diving

Several studies have reported impaired diastolic function; Martinez-Villar et al. (2022) and Boussuges et al. (2006) presented data with relaxation disturbance (Grade 1 diastolic disturbance). Castagna et al. (2017) and Marabotti et al. (2013a; b) had divers with post-dive restrictive diastology (Grade 3 diastolic disturbance). Furthermore, Hansel et al. (2012) also demonstrated restrictive diastology (Grade 3 diastolic disturbance) after simulated dry chamber dives. These studies involved shallower dives in warmer conditions than our present study.

The diastolic grade worsened in 18 (46.2%) of the divers in this study. However, most of our divers had Grade 1 diastolic disturbance after the dive versus Grade 3 diastolic disturbance, in contrast to studies by Castagna et al. (2017) and Marabotti et al. (2013a; b). It may be speculated that interplay between cold water and immersion-related hemodynamic and volume changes may have counter-acted in relation to the diastology. Diastolic grade is a dynamic phenomenon, which can be instantaneously manipulated with volume depletion (Valsalva) and volume increase (leg raise) in clinical practice (Ishizu et al. 2008; Oh et al. 2011). Volume depletion is known to improve diastolic grade, whereas volume increase worsens it. As cold-water divers were exposed to both volume depletion and volume centralization, the overall effect on the diastology was more dependent on the balance between these two effects.

To evaluate the combination of systolic and diastolic function, we calculated MPI. This variable has not been described in diving medicine before. MPI seems to be independent of heart rate, blood pressure, and age. But changes in preload among healthy individuals have an effect on MPI (Askin et al. 2023), making this variable also somewhat volume dependent. This might partly explain our finding of MPI significantly increasing.

As with the changes in systolic function in our divers, the changes in diastology were subtle and without clinical significance in healthy divers. However, in a population with pre-existing or underlying heart problems, such changes may provoke symptomatic problems during or after the dive, such as congestion or secondary arrhythmia. It would be of interest to know whether the diastolic or the systolic changes are the primary ones after a cold-water dive; but regardless, both were evident in our divers with equal potential to set off post-dive problems.

### Hemodynamic changes during cold-water dive

Diving in cold water induces hydrostatic pressure and vasoconstriction, increasing central venous return and intrathoracic blood volume accumulation, leading to increased cardiac preload (Epstein et al. 1976; Lin et al. 1984; Gabrielsen et al. 1985/1993). This relative hypervolemia in turn leads to an onset of humoral mechanisms, such as an increase in atrial natriuretic peptides and suppression of antidiuretic hormone, leading to an increased diuresis and eventually predisposing divers to dehydration (Epstein et al. 1976; Sramek et al. 2000). In concordance with the Sramek et al. (2000), Marinovic et al. (2009), and Fichtner et al. (2021) studies, the weight was reduced in all but one diver in our study. Also, the urine concentration decreased, indicating increased diuresis. Our recent publication (Piispanen et al. 2021) included 23 of the present study’s 39 divers reporting a significant skin temperature decrease during the dive. In this study, the divers were asked to assess their subjective feeling of warmth during the dive. Even though our subjects wore dry suits and thick undergarments, most of the divers experienced subjective cold during the dives. Therefore, it can be speculated that our divers were affected by both cold-water-induced hemodynamic changes and immersion-related fluid loss.

### VGE

Scuba diving, performed within no-decompression limit or with decompression demand, induces bubble formation in the majority of divers, also without any symptoms of decompression illness. The presence of VGE has been associated with cardiac changes, especially right ventricular overload and a possible impairment of ventricular relaxation (Marabotti et al. 1999, 2013a; b). The bubbles have been speculated to interact with the vascular bed via both direct mechanical effects (wedging into pulmonary capillaries) and via mediators (e.g., vasoactive substances), increasing pulmonary vascular resistance, hence affecting function of the heart’s right side (Marabotti et al. 1999, 2013a; b; Dujic et al. 2006). In the present study, we found no difference in any of the echocardiographic parameters when compared between bubblers and non-bubblers, although estimation of the pulmonary artery pressure was not performed due to focusing on other parameters during a very short time frame. All but one diver in this study showed visible bubbles in the right heart concurrently with both systolic and diastolic impairment. It is possible that even a small amount of bubbles might have an effect on the heart’s function.

### Limitations

The population consisted of a relatively homogenous healthy population, and the results may not be applicable to other populations. Although the diver population was rather large for diving medicine research, the number of the divers was limited, and a larger study group could have given a more precise understanding of the cardiac changes and their relation to other factors. We were not able to characterize systolic function using modern applications of 3D volumetry and a variety of strain measurements. Due to the short time for the post-dive cardiac evaluation, we were able to focus only on a limited number of target functions in addition to bubble measurements. In addition, the echocardiography study was performed immediately prior to and after the dive due to non-water- and non-pressure-resistant equipment, which eliminated the examination of during dive changes.

Due to extreme cold conditions, the divers used dry suits and heavy undergarments. To achieve as great an effect on the diver’s heart as possible, we conducted the study during the coldest time of the year. Similar effects apply also in warmer conditions and these results could be generalized including also wetsuit divers.

## Conclusion

This is one of the few reports of cardiac changes after a cold-water trimix CCR dive. Overall, an extremely cold-water dive induced multiple cardiac changes in both systolic and diastolic function. Such concordant changes over several parameters indicate real post-dive deterioration in cardiac function and must be recognized to avoid cardiac problems. An increasing number of divers and an increasing number of older divers as well are performing demanding technical dives. The results of this study could also be generalized to recreational dives, though the effect on divers’ bodies may not be as great as diving deep technical dives. It is essential to carefully assess the possible underlying cardiovascular risk factors in “fit-to-dive” evaluation with all divers.

### Supplementary Information

Below is the link to the electronic supplementary material.

Supplementary file1 (DOCX 26 kb)

## Abbreviations

CCR: Closed-circuit rebreather
CO: Cardiac output
EF: Left ventricular ejection fraction
IQR: Interquartile range
IVRT: Isovolumetric relaxation time
LV: Left ventricle
MAM: Mitral annulus movement
MVE Dect: Mitral declaration time
PNS: Parasympathetic nervous system
SNS: Sympathetic nervous system
RV: Right ventricle
TAPSE: Tricuspid annular plane systolic excursion
VGE: Venous gas emboli
WOB: Work of breathing
2D: Two dimensional
