# Supplementary material for: The effect of a single closed-circuit rebreather decompression dive in extremely cold water to cardiac function
Source: Eur J Appl Physiol. 2024 Jan 8;124(6):1693–702. doi: 10.1007/s00421-023-05392-0 (PMC11130038; doi:10.1007/s00421-023-05392-0)
Supplement: Supplementary file 1 — Supplementary file1 (DOCX 26 kb) [file 421_2023_5392_MOESM1_ESM.docx]

**Supplementary information**

**The Effect of a Single Closed Circuit Rebreather Decompression Dive in Extremely Cold Water to Cardiac Function**

**Echocardiographic parameters**

Examinations were made by using two-dimensional (2D) and M-mode echocardiography associated with pulsed- and continuous-wave Doppler (GE Vivid i, GEMS ultrasound, Tirat Carmel, Israel. Cardiac application module H45021JM, transducer 2D 3S-RS) with a transthoracic approach. Subjects were placed in the supine left lateral decubitus position. We made all recordings simultaneously with ECG recording in order to identify the electrical phase of each cardiac cycle. For each determinant we collected, at least seven beats and included five beats in the mean calculations. A reference examination was performed the same day 1-2 hours prior to the dive. The post-dive examination was performed at 20-40 min after the dive.

*Systolic function*

We measured left ventricle diameter during end-diastole (LV EDD) and end-systole (LV ESD) in the parasternal long-axis view. The ejection fraction (EF) was estimated by using the Teicholz formula. In addition to LV M-mode EF analysis, we also measured biplane EF values at the pre-dive phase. In onsite analysis we found that this provided no extra information to that of EF by Teicholz. Our divers were healthy, and they did not have local wall contraction abnormalities, local hypertrophy or dyssynchrony and the shape of the heart was normal. To facilitate the data collection and to ensure that the post-dive recovery phase was as short as possible, we decided to utilize the M-mode method in the follow up analyses. Left ventricular stroke volume (LV SV) was evaluated by using the diameter of left ventricular outflow tract (LVOT) and pulsed Doppler velocity time integral trace (LVOT trace). The time interval from aortic valve opening to aortic valve closure (LV ET) was evaluated with tissue Doppler imaging (TDI) below the aortic valve. Mitral annular movement (MAM) was assessed from apical four chamber view with TDI. Flow rate was calculated as LV SV / ET and the cardiac output (CO) was calculated as heart rate x LV SV.  (Lang 2015, Luis 2018, Mitchell 2019)

The assessment of right ventricular systolic function was evaluated by measuring tricuspid annular plane systolic excursion (TAPSE) in the apical four-chamber view by M-mode echocardiography and the tissue tricuspid lateral annular systolic velocity (RS’) with a tissue Doppler mode region highlighting the RV free wall. (Rudski 2010, Mitchell 2019)

*Diastolic function*

The diameter of the left atria (LA) was measured by using the maximal 2D diameter in right angle to the putative axis of the atrium. LV filling was determined by using transmitral velocities recorded by pulse wave Doppler from the apical four-chamber view: The deceleration time of early diastolic transmitral flow (MVE Dect), the peak velocity of the initial flow during rapid filling (E-wave) and the late flow (A-wave) representing the atrial contraction were recorded. The ratio of early to late transmitral flow velocities (E/A) was calculated. The mitral annular velocity (e´) was measured, and the mitral annular early diastolic velocity ratio (E/e´) was calculated. The mitral annular tissue velocity was measured in the basal septum wall. Isovolumetric relaxation time (IVRT), the interval from the aortic valve closure signal to the mitral valve opening signal was also measured. (Nagueh 2016, Oh 2011, Mitchell 2019)

As evaluation of the left ventricular diastology is a complex task that involves multiple parameters, grading of the diastology was performed according to recommendations (Ishizu 2008, Oh 2011). Diastolic grade was evaluated either as normal, as Grade 1 (relaxation disturbance), as Grade 2 (pseudonormal filling) or as Grade 3 (restrictive filling) diastolic disturbance, by using a combination of mitral inflow measurements, isovolumetric relaxation time, pulsed tissue Doppler early diastolic velocity (e’) and its ratio to mitral inflow E-wave (E/e’).

Myocardial performance index (MPI) (also known as Tei index), a ratio of time intervals, was also calculated to estimate combined systolic and diastolic function. MPI is defined as the sum of isovolumetric contraction time (IVCT) and IVRT divided by ejection time (ET). (Askin et al. 2023; Tei 1995)

**References**

1. Askin, L., Yuce, E. I., & Tanriverdi, O. (2023). Myocardial performance index and cardiovascular diseases. *Echocardiography*. https://doi.org/10.1111/echo.15628
2. Ishizu T, Seo Y, Kawano S, Watanabe S, Ishimitsu T, Aonuma K. Stratification of impaired relaxation filling patterns by passive leg lifting in patients with preserved left ventricular ejection fraction. Eur J Heart Fail. 2008;10(11):1094-101.
3. Lang RM, Badano LP, Mor-Avi V, Afilalo J, Armstrong A, Ernande L, et al. Recommendations for cardiac chamber quantification by echocardiography in adults: an update from the American Society of Echocardiography and the European Association of Cardiovascular Imaging. J Am Soc Echocardiogr. 2015;28(1):1-39 e14.
4. Luis SA, Chan J, Pellikka PA. Echocardiographic Assessment of Left Ventricular Systolic Function: An Overview of Contemporary Techniques, Including Speckle-Tracking Echocardiography. Mayo Clin Proc. 2019;94(1):125-38.
5. Mitchell C, Rahko PS, Blauwet LA, Canaday B, Finstuen JA, Foster MC, et al. Guidelines for Performing a Comprehensive Transthoracic Echocardiographic Examination in Adults: Recommendations from the American Society of Echocardiography. J Am Soc Echocardiogr. 2019;32(1):1-64.
6. Nagueh SF, Smiseth OA, Appleton CP, Byrd BF, 3rd, Dokainish H, Edvardsen T, et al. Recommendations for the Evaluation of Left Ventricular Diastolic Function by Echocardiography: An Update from the American Society of Echocardiography and the European Association of Cardiovascular Imaging. J Am Soc Echocardiogr. 2016;29(4):277-314.
7. Oh JK, Park SJ, Nagueh SF. Established and novel clinical applications of diastolic function assessment by echocardiography. Circ Cardiovasc Imaging. 2011;4(4):444-55.
8. Rudski LG, Lai WW, Afilalo J, Hua L, Handschumacher MD, Chandrasekaran K, et al. Guidelines for the echocardiographic assessment of the right heart in adults: a report from the American Society of Echocardiography endorsed by the European Association of Echocardiography, a registered branch of the European Society of Cardiology, and the Canadian Society of Echocardiography. J Am Soc Echocardiogr. 2010;23(7):685-713; quiz 86-8.
9. Tei, C. (1995). New non-invasive index for combined systolic and diastolic ventricular function. *J Cardiol*, *26*(2), 135-136. https://www.ncbi.nlm.nih.gov/pubmed/7674144
